# Features of the Territorial Distribution, Composition and Structure of Phytocenoses with the Participation of *Fraxinus excelsior*, Their Resource Qualities, Ecological and Economic Importance (Southeastern Part of the East European Plain)

**DOI:** 10.3390/life13010093

**Published:** 2022-12-28

**Authors:** Maxim Viktorovich Larionov, Alexey Anatolievich Volodkin, Olga Alexandrovna Volodkina, Evgeny Valentinovich Lebedev, Olga Evgenievna Khanbabayeva, Svetlana Vitalievna Tazina, Elena Anatolyevna Kozlova, Elena Evgenievna Orlova, Inna Nikolaevna Zubik, Varvara Dmitrievna Bogdanova, Mikhail Vladimirovich Vorobyev, Alena Pavlovna Demidova, Liliya Rafisovna Akhmetova, Yulia Igorevna Kondratenko, Ivan Ivanovich Goloktionov, Ekaterina Vladislavovna Soboleva, Karina Mikirtichevna Gordyushkina

**Affiliations:** 1 Moscow Timiryazev Agricultural Academy—Russian State Agrarian University, 49 Timiryazevskaya Street, 127550 Moscow, Russia; 2 Faculty of Ecology and Environmental Protection, Russian State Social University (RSSU), 4 Wilhelm Peak Street, Building 1, 129226 Moscow, Russia; 3 State University of Management (SUM), 99 Ryazanskij Prospect Street, 109542 Moscow, Russia; 4 Federal State Budgetary Educational Institution of Higher Education “State University of Land Use Planning” (SULUP), 15 Kazakov Street, 105064 Moscow, Russia; 5 Department “Crop Production and Forestry”, Penza State Agrarian University, 30 Botanicheskaja Street, 440014 Penza, Russia; 6 Department “Forest Inventory and Forest Management”, Nizhny Novgorod State Agricultural Academy, 97 Gagarin Avenue, 603107 Nizhny Novgorod, Russia

**Keywords:** *Fraxinus excelsior* L., range, occupied forest types, plant communities with participation of *Fraxinus excelsior* L., structure of forest phytocenoses, age spectrum of populations, the state of the populations, the need to protect ash populations, the need for afforestation based on common ash, biocenotic and economic importance, resource qualities, Volga Region, southeastern part of the East European Plain

## Abstract

At present, the distribution area of *Fraxinus excelsior* L. in the forest ecosystems of the Volga Region is rather low and ranges from 0.01% to 2.5%. In the Middle Volga Region, using the example of the Penza region, five types of deciduous forests were identified in the composition with *Fraxinus excelsior* L.: oak forest aegopodium, oak forest nettle, oak forest hazel-linden, oak forest aegopodium-motley grass, oak forest carex-motley grass. In the forest phytocenoses of the Moksha River basin, the quality of *Fraxinus excelsior* L. is 1.5–1.7. In the forest phytocenoses of the Khoper River basin, the average quality value reaches 2.4–2.8, and in the forest tracts of the Sura river basin it is 2.8–3.2. In the western part of the study area, individuals of age class II–III (21–40, 41–60 years) predominate, in the central part—age class I (1–20 years), in the eastern part—age class V (81–100 years). This circumstance allows us to conclude that its populations in the western regions are represented by stands of different ages; the presence of young stands and middle-aged stands indicates the presence of conditions for reproduction and distribution. At the border of its range, *Fraxinus excelsior* L. grows in a stable population; in the western part of the Middle Volga Region, the number of species in forest stands with a predominance of *Fraxinus excelsior* L. is 26–30% higher than this indicator in more eastern regions. In the direction from east to west, the number of species in the composition of forest stands increases (up to 8.4), with a predominance of *Fraxinus excelsior* L. The number of plant associations increases in the direction from east to west. If in the east of the Penza region *Fraxinus excelsior* L. occurs in 6–7 plant associations, then in the west of the region—in 18–25 associations. The maximum timber stock for 100 years of *Fraxinus excelsior* L. stands reaches 380 m^3^/ha. Such a natural bioresource potential is of importance for the conduct of the national economy. Forest management in phytocenoses with the participation of this tree species is a strategic branch direction. It is expedient to restore populations of *Fraxinus excelsior* L. everywhere and to cultivate them in the territory of the East European Plain and especially in its south-eastern part. This is fully consistent with the principles of sustainable ecological and economic development against the background of local natural, climatic and geographical conditions. This type is necessary when solving environmental, resource-saving and economic problems in the territory under consideration.

## 1. Introduction

*Fraxinus excelsior* L. plays an important role in ensuring sustainability and increasing the biodiversity of forest ecosystems in western [1,2,3], northern [4], southern [5] and eastern Europe [6,7]. This fully applies to the territory of the East European Plain [8,9,10]. This role is realized by providing and increasing the species and ecological diversity of forest stands in natural and natural-economic plant communities. This species rarely forms pure stands. Phytocenoses with its participation are represented by multispecies associations of different representatives of woody plants and are distinguished by high species diversity and productivity. This has been noted for many areas of Europe [11,12,13,14]. It is noted that this species is characterized by good survival [15] and rapid growth [16].

On the territory of the East European Plain, *Fraxinus excelsior* L. is often included in forest stands together with *Quercus robur* L., *Acer platanoides* L., *Tilia cordata* Mill. and *Populus tremula* L. [17,18]. It grows in forests on dark gray loams and podzolized chernozems. Also, individuals of this species are often found in the phytocenoses of river floodplains. It is distributed in beam (bayrach) forests located along a beam network (beams, dry valleys, hollows), in the forest–steppe zone and in the northern part of the steppe natural zone. *Fraxinus excelsior* L. also inhabits forest edges [19,20]. In Europe, individuals of this species also prefer fertile and at the same time freely drained soils. On such soils, they form dense woody thickets [12,19].

Mixed stands of *Fraxinus excelsior* L., especially with an admixture of broad-leaved tree species (oak, maple, linden), have a better phytosanitary condition compared to stands of other representatives of deciduous woody plants that are pure in composition [21,22,23]. This species is involved in the formation of the vertical, horizontal and ecological structure of plant communities [23,24,25,26,27,28,29,30,31]. It largely determines the biological and ecosystem diversity of natural landscapes.

As a crop for urban and suburban landscaping, *Fraxinus excelsior* L. in the Middle and Lower Volga Regions, and in a number of regions of the Chernozem Region, did not receive due attention [32,33,34]. This is observed everywhere within the settlements of the Volga and Chernozem Regions and in their immediate vicinity [34,35,36,37,38,39,40]. Unfortunately, this trend continues to the present moment, despite the adaptability of the species to local natural–geographical and ecological conditions.

Traditionally, this species in cultivation in Eastern Europe and, in particular in the European part of Russia, has earned the most attention in forestry. Common ash has unique biological, ecological and resource properties [39,40,41,42,43].

The range of *Fraxinus excelsior* L. is quite extensive; it stretches in the meridional direction from west to east, from the shores of the Atlantic Ocean to the Volga River in Russia. It is a widespread breed that grows throughout Europe. Plantations of *Fraxinus excelsior* L., growing in the western part of the range, have recently suffered greatly from damage by pests and diseases. In the eastern part of the range in the study area, plantations of *Fraxinus excelsior* L. are resistant to damage by biotic pathogenic factors and increase the biodiversity of broad-leaved forests, but the species is not able to spread further eastward, to the territories of the Trans-Volga Region.

The authors set a goal to study the features of the biogeographic distribution of phytocenoses with the participation of *Fraxinus excelsior* L. in the European part of Russia, the features of their composition and structure, confinement to certain habitat conditions, as well as the resource qualities and ecological and economic importance of its plantations on the eastern border to establish the factors limiting its further distribution in the eastern direction.

## 2. Materials and Methods

Our study was carried out on the territory of the Middle Volga Region, located in the east of the European part of Russia (from 42° to 47° east longitude and from 54° to 52° north latitude), in a temperate geographical zone, at the junction of forest, forest–steppe and steppe natural zones. The vast part of the territory is occupied by the western slopes of the Volga Upland; the extreme west belongs to the eastern outskirts of the Oka–Don Plain. The region has a temperate continental climate [44,45].

Vegetation cover is characterized by a significant diversity; a third of the territory has preserved native vegetation cover. Forest areas are represented by broad-leaved communities (oak forests), coniferous communities (pine and birch–pine forests) and small-leaved communities (aspen and birch forests).

The mean annual temperature (MAT) is 5.2 °C; the mean annual rainfall (MAP) is 563 mm. The annual duration of sunshine is 1800 h; the average annual relative humidity is 75.0%.

The research work was carried out in three stages. The first, preparatory stage, was implemented in office conditions. For the assessment, we used the data of the inventory of the forest fund [46]. The watershed lines of the Moksha, Sura and Khoper rivers were chosen as the boundaries of the forest-growing areas. The boundaries were drawn according to the topographic map M 1:50.000. The main unit of the study was quarters. Individual quarters of the forest fund were assigned to one or another basin. The composition of plantings was determined according to the data on timber stocks by constituent species for each quarter. The analysis of the composition of forest stands was carried out using the Excel spreadsheet system. In the analysis of taxation characteristics, mass materials of the forest inventory of 2005 were involved. The entire array of the data of taxation descriptions is divided into forest types accepted for the territory of the Volga Region (for example, the Penza region).

The second stage, field research and observations. Plantations of *Fraxinus excelsior* L. were selected in 5 types of forests and types of habitat conditions. The share of participation of each species was determined based on its presence in the composition of the forest stand (according to the formula of the composition of the forest stand). The degree of distribution of an individual species in the composition of plant associations was estimated by the number of trees of the species, depending on the age, relative density and share of their participation in the composition of forest stands, as well as the area it occupied relative to the total area of forest stands of each type of forest. Based on the same methodological approaches, the participation of each species in the stands of various forest types was also calculated, while for each gradation of the composition of the stand (10%), the corresponding part of the area of distribution of the species was indicated in % of the total area. The role of species was assessed both by their distribution over the area and by the degree of participation in the composition of the forest stand. Such an approach to assessing the cenotic significance of a species in the composition of forest stands makes it possible to assess the nature of their behavior in various types of forests, as well as to trace changes in forest communities over time [47,48]. Thirty sites were selected for each type of forest at different ages according to age classes—young, middle-aged, maturing, mature and overmature [49,50].

The concept of a forest type as a forest area or their combination characterized by common types of forest conditions, the same composition of tree species, the number of tiers, similar fauna, requiring the same forestry measures under equal economic conditions, is given according to the definition of V.N. Sukachev [51,52].

The name “oak forest” in the forest types oak forest aegopodium, oak forest nettle, oak forest hazel-linden, oak forest aegopodium-motley grass, oak forest carex-motley grass indicates that the main species in plant associations is *Quercus robur* L.; the second characteristic indicates the main plant prevailing in the herbaceous ground cover of the stand (*Aegopodium podagraria* L., *Urtica dioica* L., *Carex pilosa* Scop.) or the dominant species of associated tree species (*Corylus avellana* L., *Corylus avellana* L.). Types of forest conditions, C2 (fresh sudubrava), D1 (dry oak forest), D2 (fresh oak forest), D3 (moist oak forest), are given according to the classification of P.S. Pogrebnyak [52,53].

Taxation indicators are given according to the main characteristics used in forest inventory in the course of forest management in the territory of the Russian Federation [54]. To characterize the productivity of *Fraxinus excelsior* L. forest stands, we used the conditional indicator of forest bonitet (German Bonität, translated from Latin bonitas—good quality, high quality [43,47]). The indicator is determined by the average height and age of the tree species. *Fraxinus excelsior* L. in the conditions of the Middle Volga Region reaches an impressive size and structure of the crown, as in the western parts of its range. High quality (high quality) stands are considered to be I and II classes of quality, low quality (low quality) stands IV and V classes of quality.

Trial plots were established in plantations of *Fraxinus excelsior* L. of different ages, the sample size was 0.04 ha, and a complete enumeration of trees was carried out. The diameter of the trees on the trial plots was measured with a measuring fork, and the height with a forest altimeter.

The third stage is statistical analysis. At the end of the field research, an analysis of the obtained data was carried out. The average values of forest stands were established in terms of height, diameter, volume of wood in various parts of the study area, in various types of forests and types of forest growth conditions.

## 3. Results

The study area has natural, climatic and soil conditions that are generally typical for the Middle Volga Region, which belongs to the territories of the temperate climate zone with dry, hot summers and cold winters with precipitation in the form of snow. In the summer, western air currents prevail in the territory. From October to April, Asian air currents are the climate-forming factor. Soils in the western and central regions of the study area are represented by dark gray forest, gray forest soils and podzolized, clayey and heavy loamy chernozems, while in the eastern region they are represented by light gray and gray forest soils, medium loamy and light loamy [44,45].

The range of *Fraxinus excelsior* L. is much smaller than that of *Quercus robur* L. It is distributed throughout Western and Eastern Europe [24,25], in the Mediterranean [26] and Asia Minor, in Russia—south and west of the Volga river, in the Caucasus [27,28]. The northern border of the range runs from St. Petersburg to the southeast. Further, it crosses the Volga river below Kostroma, passes to the right bank of the Volga river and goes along it to the city of Nizhny Novgorod. Then, the border of the range turns to the east and, strictly adhering to the northern slopes of the Volga Upland, follows to Kazan. Turning south, the range of *Fraxinus excelsior* L. reaches the Sura river and continues south to Penza. Then, the boundary of the range of this species goes along the banks of the Volga river to Volgograd. From Volgograd, the boundary of the species follows the mouth of the Medveditsa river, and then through Rostov oblast it leaves Russia [29,30]. In the territory of the Penza region, it is located on the eastern border of its natural range [31,32]. In the Lower Volga Region, this species has a rare distribution. It is confined mainly to small forests in the forest–steppe and steppe zones, as well as in urban and suburban conditions due to landscaping.

*Fraxinus excelsior* L. grows mainly in mixed stands. Less often, pure stands or stands with a predominance of this tree species are formed. Its relatively high shade tolerance in the juvenile period and ecological plasticity allow it to form a population in the forest association with *Quercus robur* L. In a significant number of cases, *Fraxinus excelsior* L. grows in oak forests, one of the main species in the forest stand. It is also able to renew itself both by seed and by coppice [41,42].

The analysis of forest registry data for regions located on the East European Plain showed a significant difference in the areas of territorial distribution of *Fraxinus excelsior* L. (Table 1).

The study of the distribution of *Fraxinus excelsior* L. in the eastern part of its range by analyzing its share in forest plantations of the Middle and Lower Volga Regions, on the territory of the subjects on the right bank of the Volga River, shows that in the territory of the Samara Region, forest stands with its predominance in the composition grow on 2.5% of the forested area, in Penza on 0.5%. In the Nizhny Novgorod Region, it is distributed only on the Volga–Oka Right Bank (0.02%). On the territory of the Republic of Tatarstan in the Volga Region, it occupies small areas (0.03%). In the Ulyanovsk Region, its presence in the composition of broad-leaved and coniferous-broad-leaved forests (0.01%) is practically uncharacteristic. The conducted studies showed that the representation of *Fraxinus excelsior* L. specimens in the forest ecosystems of the Volga Region is quite low and ranges from 0.01% to 2.5%.

**Figure 1:**
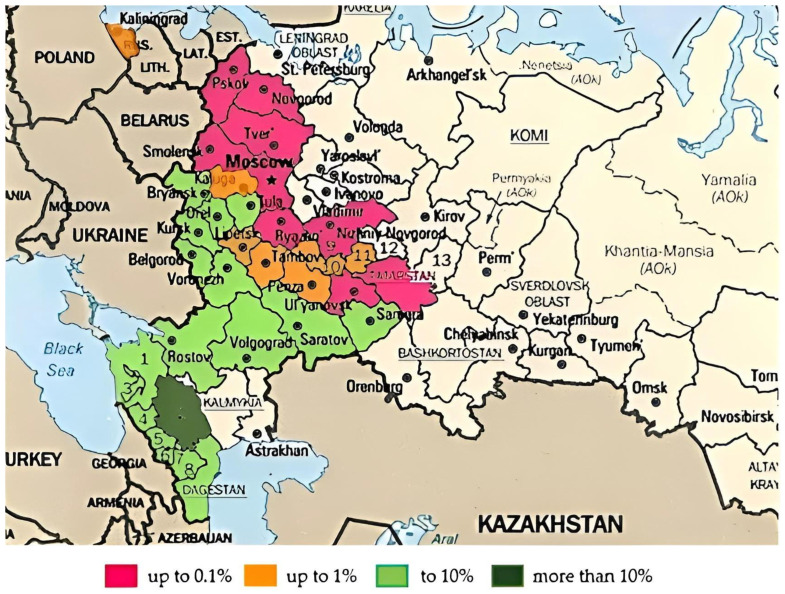
Percentage of forests dominated by *Fraxinus excelsior* L. in the forested area, %. 1—Krasnodar Territory; 2—Stavropol Territory; 3—Adygea, 4—Karachaevo–Cherkessia; 5—Kabardino–Balkaria; 6—North Ossetia; 7—Ingushetia; 8—Chechen Republic; 9—Nizhny Novgorod Region; 10—Mordovia; 11—Chuvashia; 12—Mari El; 13—Udmurtia.

In the more southern regions, the proportion of plantations of *Fraxinus excelsior* L. turned out to be higher than in the northern ones. At the same time, its largest areas in the Lower and Middle Volga Regions were recorded in the Samara, Saratov and Volgograd Regions. Its distribution to the Trans-Volga territories of the largest river in Europe is difficult due to the presence of a wide sandy strip along the banks of the river (Figure 1).

Shustov, V.S. [39] pointed out that the distribution of the fruits of the *Fraxinus excelsior* L. in open areas does not exceed 180–200 m. Through this, its natural distribution by seed cannot occur. The distribution of *Fraxinus excelsior L.* fruits in open areas does not exceed 180–200 m. In the forests, its fruits are carried to a distance within 2–3 heights of the mother trees. The obstacles to its spread are not only mountains, rivers, etc. In addition, significant barriers are open spaces over 200 m. East of the floodplain of the Volga River, *Fraxinus excelsior* L. does not grow in its natural state. This can be explained by the following reasons: the nature of the soil cover of the Volga Upland and the increase in the continental climate, characterized by frequent spring frosts and the early onset of morning frosts in autumn. In fact, in the Orenburg region, located east of the Volga River, late spring frosts can be observed even in early July, and autumn frosts begin as early as August, which limits the possibility of its spread to the region. This is also indicated by A. Yu. Kudryavtsev [40]. High air temperatures in the summer months do not adversely affect its growth in the southern regions of the European part of Russia with a continental climate, where it occupies significant areas ranging from 4 to 26% of the forested areas.

An additional limiting factor in the spread and increase in *Fraxinus excelsior* L. planting areas is the reduction in the area of *Fraxinus excelsior* L. forest plantations, which is also confirmed by our research.

*Fraxinus excelsior* L. prefers moist, fertile, acid-neutral soils; being a heat-loving and light-loving plant, it forms forest stands along river floodplains, in ravines and natural landscape depressions. Research by A. Yu. Kudryavtsev found that as the aridity of the climate increases, it passes into more humid ecotopes.

The conducted studies have shown that under favorable conditions for its development, corresponding to the biological characteristics of this tree species, in the conditions of the Middle and Lower Volga Regions, *Fraxinus excelsior* L. forms stable and highly productive stands.

It was established that in the basin of the Moksha river, located in the western part of the study area, the average bonitet of *Fraxinus excelsior* L. plantations is high and varies from I.5 to I.7, decreases from II.4 to II.8, in the eastern part of the region and in the Sura river basin significantly decreases from II.8 to III.2. On the eastern border of its range, it has perfectly adapted to the conditions of the region and forms plantations with a significant average height and diameter, but an order of magnitude lower than in its more western parts.

In the conditions of the Volga Region, individuals of this species are found in complex oak forests and in simple forest communities. In oak forests, along with *Quercus robur* L., its companions grow: *Acer platanoides* L., *Tilia cordata* Mill., *Ulmus minor* Mill., *Ulmus laevis* Pall., *Ulmus glabra* Hudson and others. The biocenotic interactions between the woody plants are very complex.

*Fraxinus excelsior* L., *Quercus robur* L., *Populus tremula* L. and *Betula pendula* Roth dominate in the composition of the oak forests in the first layer. *Tilia cordata* Mill, *Acer platanoides* L., *Acer campestre* L., *Corylus avellana* L. predominate in the second tier of forest phytocenoses. Actually, these woody plants form the core of the species spatial and ecological structure of the forest communities in the Volga Region. That is, *Fraxinus excelsior* L. (along with *Quercus robur* L.) is a forest-forming species. It forms the specific, vertical and to a large extent horizontal structure of phytocenoses in the forest–steppe zone of the Middle Volga Region. Similar surveys of forest ecosystems in the forest–steppe and steppe zones of the Saratov region and in the steppe zone of the Volgograd region (Lower Volga Region) also confirmed this conclusion. Fraxinus excelsior L. together with *Quercus robur* L. form forest communities and optimizers on the watershed landscapes of the Volga regions.

The vital state of *Fraxinus excelsior* L. plants in most forest areas turned out to be good (forests remote from urban settlements and intercity highways) and at the level of slight weakening (forest areas in suburban areas).

Long-term surveys on large areas of forests in the Saratov and Volgograd regions show that the average generative individuals of *Fraxinus excelsior* L. are in a good and ecologically acceptable life condition; in forests and forest plantations near cities and large settlements, their living condition is less than normal by an average of 15–25%.

When conducting the analysis, the system of ranking the age of plantations by age classes adopted in Russian legislation for hardwood species was used. Each age stage in hardwood associations formed by *Quercus robur* L., *Fraxinus excelsior* L. and *Acer platanoides* L. is taken to be equal to 20 years. The age of the plantations was determined according to the materials of the plantation inventory, as well as using an age borer on the test plots.

The analysis of the age structure of the populations of tree species with a predominance of *Fraxinus excelsior* L. in the Volga Region (on the example of the Penza region), made for convenience by age classes of the plantations, showed the following. In the western part of the study area, individuals of age class II–III (21–40, 41–60 years) predominate, in the central part—age class I (1–20 years), in the eastern part—age class V (81–100 years). This circumstance allows us to conclude that its populations in the western regions are represented by stands of different ages; the presence of young and middle-aged stands indicates the presence of conditions for reproduction and its distribution. The young and middle-aged stands have a high growth and seed productivity; the population is able to continue its further existence because, due to the presence of groups of different ages in the population, its ability to withstand the influence of the environment is increased.

In old stands, growth in height and diameter is weakened, reproductive capacity is reduced and resistance to abiotic and biotic environmental factors is reduced. These data are reflected in Table 2.

In general, the distribution of forest stands by age is uniform, which allows us to conclude that *Fraxinus excelsior* L. grows in a stable population at the border of its range.

In the western part of the Middle Volga Region, the number of species in forest stands dominated by *Fraxinus excelsior* L. is 26–30% higher than in the more eastern regions.

Thus, in the direction from east to west, the number of species in the composition of forest stands increases with the predominance of *Fraxinus excelsior* L. This is also evidenced by the average number of species—up to 8.4. This indicator is characteristic of the westernmost part of the study area, characterized by a large amount of precipitation, a decrease in the continentality of the climate and richer soil nutrients.

Studies have found that *Fraxinus excelsior* L. grows in mixed deciduous plantations (Table 3). The composition of codominants of forest phytocenoses in the Volga Region includes *Quercus robur* L. (first of all), *Acer platanoides* L., *Populus tremula* L., *Betula pendula* Roth, *Tilia cordata* Mill, *Salix fragilis* L., *Populus balsamifera* L. As a result of the interaction of tree species, there are various complex plant associations.

The general trend in the structure of forest stands with the predominance of *Fraxinus excelsior* L. refers to the type—seven to ten mixed, complex species of tree associations with undergrowth and undergrowth. A plant association is a set of homogeneous phytocenoses, the composition of which is the result of the interaction between all the components of the biogeocenosis and environmental conditions [47,51].

The composition of woody vegetation is closely related to the abiotic and biotic environmental conditions of the area: soil, climate, representatives of flora and fauna. These conditions and the composition of tree species associations are fully characteristic of the south-eastern part of the East European Plain.

**Figure 2:**
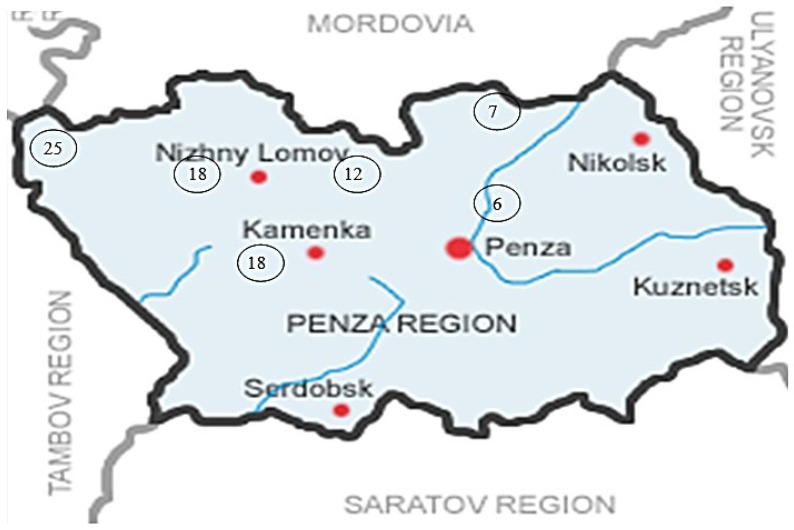
The number of plant associations in communities formed by the stands of *Fraxinus excelsior* L.

The number of plant associations increases in the direction from east to west. If in the east of the Penza region *Fraxinus excelsior* L. occurs in 6–7 plant associations, then in the west of the region—in 18–25 associations. The representation of *Fraxinus excelsior* L. in different types of forest changes in the same way. Oak forest types are developed in the western part of the region. The habitat conditions of *Fraxinus excelsior* L. are very diverse. The forest communities with the participation of *Fraxinus excelsior* L. in the westernmost part of the region are characterized by the highest biodiversity, forming 25 different types of plant associations. Complex plant associations are formed in forest types characterized by rich trophotopes—oak forests and sudubraves and hygrotopes with fairly high moisture (Figure 2).

Various varieties of associations with a predominance of *Fraxinus excelsior* L. in the dominant layer constitute a group of associations. They are distinguished by biodiversity; they are the basis of forest biogeocenoses. In general, forest tree species (including *Fraxinus excelsior* L.) spread across the east and southeast of the East European Plain. This is the most important condition for the conservation and development of natural forests. This circumstance is characteristic of all forest ecosystems in the forest–steppe and steppe zones of the Volga Region.

Forest stands of *Fraxinus excelsior* L. are recorded in five types of oak plant associations. Upon reaching the second class of age, an individual of *Fraxinus excelsior* L. in the oak forest type of aegopodium-motley grass has a wood reserve of 150 m^3^/ha, in the hazel–linden forest type 140 m^3^/ha, in the aegopodium forest type 124 m^3^/ha, nettle forest type 130 m^3^/ha, carex-motley grass forest type 80 m^3^/ha.

To determine the highest productivity of *Fraxinus excelsior* L. plantations, the average annual growth by forest type was determined, which made it possible to compare the intensity of the growth and development of stands. The results are presented in Table 4.

The maximum stock of wood from *Fraxinus excelsior* L. forms in the forest type oak aegopodium, the type of growing conditions—fresh oak forest (D2). At the age of 100 years, the stock in the ash stand is 380 m^3^/ha. The annual growth is 3.8 m^3^. Also in the forest type oak forest, the nettle type of habitat condition is damp oak forest (D3). The stock in the stands of *Fraxinus excelsior* L. at the age of 100 years is 369 m^3^ per 1 ha. The annual growth is 3.69 m^3^.

The annual increase in height in different types of forests does not have significant differences (Table 5). The annual increase in diameter depends on soil fertility and moisture conditions. Mature trees are considered to be of all grades from 101 years old. In this regard, we can conclude that mature and overmature stands make up a small area. The largest area is made up of young stands of I and II age classes: 48% of the total area of *Fraxinus excelsior* L. forest stands. This indicates a good renewing capacity of the natural and artificial forest stands in the conditions of the Middle Volga Region. In the Lower Volga region, the regeneration capacity of *Fraxinus excelsior* L. in forest ecosystems is 10–20% lower.

*Fraxinus excelsior* L. trees reach the highest average height and average diameter in stands with nettle oak forest types. In such arrays, with the type of growing conditions—fresh oak forest (D3): 16.3 m and 18.2 cm, respectively, the wood reserve is 161.0 m^3^. The average indicators of the destructive size of trees in the study area are 14.6 cm in height, 16.2 cm in diameter and the stock is 143.3 m^3^. The density of plantations reflects the density of trees and the space they occupy. In our work, we used an indicator of the relative density of plantings, in comparison with reference plantations and expressed in fractions of a unit (0.1 … 1.0). The relative density of the forest stands is average and amounts to 0.7.

Considering the value of *Fraxinus excelsior* L. wood and the high total bioecological potential of its forest stands, it is necessary to increase the area of ash forest stands in the conditions of the Middle Volga Region. A similar conclusion applies to the territories of the Lower Volga Region and Chernozem Region. It is quite realistic to implement this by creating forest plantations of *Fraxinus excelsior* L. on clearings of low-value plantations of softwood species. A large territorial resource for afforestation through the cultivation of *Fraxinus excelsior* L. in the forest–steppe and steppe regions of the Volga Region is available in the unused formerly agricultural, urban, transport-economic and technogenic-production lands of the region, alongside waste land, reserve land and territories of soil and water protection. The individuals of the *Fraxinus excelsior* L. in the immature and generative age stages are able to form highly productive and highly ecologically significant forest plantations. These bio-ecological qualities of *Fraxinus excelsior* L., aboriginal for the forest–steppe and steppe ecosystems of the Volga Region, are useful everywhere in afforestation, reforestation, agricultural and forestry reclamation and in the ecological rehabilitation of wasteful, inconvenient and economically unsuitable land.

*Fraxinus excelsior* L. trees must also be left in forests of natural origin and in forest plantations during maintenance felling, in the plantations of other tree species. This has the ability to give sustainable natural renewal in the conditions of the forest–steppe of the Middle Volga Region and the forest–steppe and steppe of the Lower Volga region. Thus, the stands of this species are an important ecological factor in ensuring the sustainability of the forest (and urban) ecosystems in the regions of the Volga Region. In order to increase the coenotic significance of *Fraxinus excelsior* L. in the conditions of the Volga Region, it is necessary to increase the area of growth of the individuals of this species in various territories through large-scale regional and interregional afforestation projects, suburban and urban gardening and the creation of a sustainable transport system and landscape-optimization gardening, silvicultural production and the creation of dendrological nurseries and specialized dendrological gardens. Nurseries with cultures of woody plants from local (natural) phytocenoses are especially required as a kind of uterine center (for preparing planting material) in areas remote from regional centers in the Penza, Saratov and Volgograd regions and in some other regions. Moreover, the latter are urgently needed in various Volga Regions and in other territories of the East European Plain.

A significant role should be given to the implementation of measures to promote the natural regeneration of *Fraxinus excelsior* L. in the various forest conditions of the Volga region, which are most favorable for normal growth and development. Areas in natural forests with natural regeneration and areas with individuals of *Fraxinus excelsior* L. in the initial stages of development should be subject to regulatory control. This applies to both forests of natural origin and forest plantations of various economic categories. Without the direct intervention of municipal and regional authorities, forest protection measures will be ineffective.

## 4. Discussion

We studied the distribution of *Fraxinus excelsior* L. stands in the eastern part of their range on a large regional scale in the Middle Volga. These studies show that the diversity of the structure and composition of mixed plantations, their habitual sizes and the spatial pattern of their distribution are associated on a large scale with soil conditions in forests and weather patterns in the early spring and late autumn periods of forest stand growth.

In the Volga Region, the most significant areas of natural forests with the predominance of *Fraxinus excelsior* L. and with their presence in mixed forest stands are located on the territory of the forest fund. The forest phytocenoses with the participation of *Fraxinus excelsior* L. in the western part of the study area are characterized by the highest biodiversity. Here, stands of this species form 25 different types of plant associations. The number of plant associations increases in the direction from east to west; if in the eastern part of the Volga Region it occurs in 6–7 plant associations, then in its west—in 18–25 associations. In the same dependence, its representation in various types of forest also changes. The types of growing conditions for *Fraxinus excelsior* L. differ in significant diversity. A similar trend is also characteristic of the north of the Lower Volga Region and the southeast of the Central Chernozem Region.

Upon reaching the second class of age, an individual of *Fraxinus excelsior* L. in the oak forest type of aegopodium-motley grass has a wood reserve of 150 m^3^/ha, in the hazel–linden forest type 140 m^3^/ha, in the aegopodium forest type 124 m^3^/ha, nettle forest type 130 m^3^/ha, carex-motley grass forest type 80 m^3^/ha. It forms the maximum wood reserve in the forest oak type of forest: the type of growing condition is fresh oak forest. At 100 years, the stock in stands of this species is 380 m^3^ / ha. The annual growth is 3.8 m^3^. The impressive resource qualities of *Fraxinus excelsior* L. forest stands also determine the corresponding resource and economic potential of forest phytocenoses for the development of national economy areas based on the consumption and reproduction of the bioresources of such communities.

It is necessary to expand the areas of the forest plantations of *Fraxinus excelsior* L. in different conditions of habitats. It is also advisable to carry this out in areas unsuitable for agriculture and urban economy, including in waste areas.

The development of relatively stable populations of the considered species and, on its basis, phytocenoses in various territories has been noted [55,56,57,58,59]. For the study area and for the entire East European Plain, reforestation, afforestation and silvicultural production based on the planting of *Fraxinus excelsior* L. is an important practical task.

This will increase the species, the genetic and ecological diversity of the local ecosystems and the biological productivity of the forests and plantations. As a result, this work will improve and ensure the environmental sustainability of forest ecosystems in areas remote from settlements and urban ecosystems in the Volga Region.

Taking into account the bioecological qualities of *Fraxinus excelsior* L., useful in resource [60,61] and biocenotic planning [62,63], it is advisable to use it everywhere in reforestation, in silvicultural production and in landscaping the adjacent and internal territories of settlements. The expansion of forested areas on the basis of the considered species in the East European Plain allows the solving of many economic issues.

In relation to Fraxinus excelsior L. and other native tree species in natural and artificial ecosystems, it is objectively required to ensure constant phytosanitary and ecological monitoring [64,65,66,67,68,69,70,71]. Different types of monitoring [71,72,73,74,75,76,77,78,79,80,81] should not be local. The phytosanitary and ecological monitoring of the common ash and related woody plants in phytocenoses should be implemented throughout their natural range and culture. Monitoring the state of forest stands in natural and artificial ecosystems can form the biological and ecological basis for the creation of a unified database of environmental data in the field of the rationalization of bioresource use, nature conservation, agricultural and forest reclamation and in the development of highly efficient environmental resource management.

Given that this is a native and highly productive species, its introduction into the culture also eliminates many negative phenomena in terms of unwanted plant invasions. It is extremely important to ensure this in relation to forest phytocenoses adjacent to the created artificial phytocenoses in the suburban, urban and rural areas of the Volga Region.

The expediency of protecting populations of this species can be traced in different parts of the range of the species under consideration [82,83,84,85]. The protection of the existing forests with the participation of *Fraxinus excelsior* L. in the south-eastern part of the East European Plain is a strategic ecological and economic task. It is also obvious that there is a need to create breeding nurseries for the conservation of genetic diversity and the selection of an environmentally sustainable planting assortment using the example of *Fraxinus excelsior* L. from different parts of its distribution area. This is due to a large area of distribution, a variety of weather and climatic conditions, the biology of the species and the unique features of the formation of multi-species plant associations with the participation of this species.

In many ways, the organizational and ecological functionality of forests in this area depends on the ability of this species to renew and spread. The established resource qualities of *Fraxinus excelsior* L. stands in the investigated types of forests of the Volga Region represent potential ecosystem services for many branches of the national economy. This is especially true for the areas of production and construction related to forest management and dependent on it. Afforestation based on *Fraxinus excelsior* L. makes it possible to ensure the ecological stability of natural landscapes, agricultural and urban natural and economic complexes. Forests and forest belts based on this species are of great importance for agricultural and forest reclamation. And this applies to natural and artificial forest ecosystems. Near forests and forest belts with the participation of *Fraxinus excelsior* L., the level of carbon sequestration in soils is 25–40 percent higher compared to open spaces. In agricultural soils, the content of organic matter is higher on average by 20–35 percent. The physical, chemical and biological characteristics of soils are improved. This is constantly ensured due to the favorable ecological impact on soils and landscapes of phytocenoses based on *Fraxinus excelsior* L. Due to the specific features of the biology and ecology of this species, it is possible to control the succession processes, structure, condition and environment-forming role of forest ecological systems. There was no need to build a mathematical model to analyze the distribution and place in the biogeocenoses of this tree species.

## 5. Conclusions

The widespread creation of forest plantations in this area based on the planting of *Fraxinus excelsior* L. trees will allow the formation of sustainable ecological frameworks for local natural territorial complexes. The bioecological properties of the species allow this on the territory of the Volga Region. Such work will help maintain the structure, species composition and normal ecological state of the forest ecosystems and neighboring meadow, steppe and river ecosystems. This work is especially needed in the Middle and Lower Volga Regions, in the central region and in other areas of the East European Plain. The widespread cultivation of forests based on *Fraxinus excelsior* L. on the territory of the East European Plain fully meets the principles of sustainable ecological and economic development. This native species for this territory is able to perform both natural biocenotic functions and economic tasks. The natural qualities of *Fraxinus excelsior* L. allow the species to realize its bioecological potential in the natural, climatic and landscape conditions of the Volga Region and a number of other subregions of the East European Plain.

In turn, such tasks are quite feasible in forest management, in protective landscaping around and within agricultural lands, in agricultural and forest reclamation and in suburban and urban landscaping. These practical tasks are of particular importance against the background of the deterioration of the soil conditions and the weakening of the ecosystems in the suburban and urban areas of the Volga Region, the central region and a number of other regions of the East European Plain [46,47,48,49,50,51,52]. With the protection and widespread distribution of *Fraxinus excelsior* L. in the culture, it is possible to implement rational resource use and renew the bioresource potential of the forest, forest–steppe and steppe ecosystems.

The conducted studies have revealed patterns in the distribution of ash in the European part of Russia. At the same time, the main limiting factor is the provision of soil with moisture in the root layer of the soil. As the depth of groundwater occurrence increases, the areas of ash plantations are reduced. The second factor limiting the distribution of ash is soil conditions. As the areas of land occupied by sandy and sandy loam soils increase, which is typical for the territories of the Middle Volga and Trans-Volga Regions, a decrease in the area of ash advancement in the eastern direction is observed. The spread of ash in the northern regions of Russia is restrained by a short growing season (with an average air temperature above 10 °C), as well as frequent late spring and early autumn frosts, and in the south, it is limited by long dry periods with high air temperatures in the summer months. Thus, the natural distribution of common ash and the increase in its range are closely related to environmental abiotic factors. The ash prefers moist areas with a temperate climate and fertile soils that can retain moisture. Such habitat conditions represent a rather narrow niche, which causes the mosaic nature of the ash area and limits its distribution.

## Figures and Tables

**Table 1:** Areas of distribution of *Fraxinus excelsior* L. in the south-eastern part of the East European Plain.

Name of Regions	Forest Area Dominated by *Fraxinus excelsior* L., Thousand ha	Areas Covered with Forests, Thousand ha	%
Volga Region (listing regions from north to south)			
Nizhny Novgorod Region	0.7	3505.4	0.02
Chuvash Republic	3.1	555.8	0.6
Republic of Tatarstan	0.4	1188.6	0.03
The Republic of Mordovia	6.3	643.0	0.97
Penza region	4.4	862.9	0.5
Ulyanovsk region	0.1	914.8	0.01
Samara region	13.5	536.1	2.5
Saratov region	24.4	577.4	4.2
Volgograd region	30.4	461.4	6.6
Northern part of the East European Plain			
Pskov region	0.1	2074.7	0.005
Novgorod region	0.1	3354.9	0.003
Tver region	0.2	4398.4	0.002
Smolensk region	1.3	1743.8	0.07
Central part of the East European Plain			
Moscow region	0.3	1738.6	0.02
Ryazan Oblast	0.2	789.9	0.02
Tambov region	0.5	374.8	0.1
Lipetsk region	1.6	160.0	1.0
Southern part of the East European Plain			
Voronezh region	19.5	389.3	5.0
Rostov region	14.9	213.9	7.0
Stavropol region	24.4	92.1	26.5
Western part of the East European Plain			
Kaliningrad region	1.3	238.4	0.55
Oryol Region	2.0	95.8	2.1
Belgorod region	13.8	219.7	6.3

**Table 2:** Distribution of *Fraxinus excelsior L.* forest areas by age classes in the Volga Region, %.

Study Area	Distribution of Planting Area by Age, %						
	I (1–20 Years)	II (21–40 Years)	III (41–60 Years)	IV (61–80 Years)	V (81–100 Years)	VI (101–120 Years)	Total
East End	25	26	14	3	32	–	100
Central part	39	22	17	16	5	1	100
Western part	23	29	30	5	13	–	100
Regional average	26	22	23	22	7	–	100

**Table 3:** Species composition of forest stands dominated by *Fraxinus excelsior* L. in the Volga Region.

Research Area	Average Number of Species	Average Share of Participation of Species in Forest Stands, %							Occurrence of Species in Communities
		*Fraxinus excelsior*	*Quercus* *robur*	*Acer* *platanoides*	*Tilia* *cordata*	*Betula* *pendula*	*Populus* *tremula*	Others	
Eastern District	2.8	34	8	8	28	8	3	10	7
Central District	5.8	35	18	17	17	5	7	6	8
Western District	7.2	30	14	16	13	9	9	8	10

**Table 4:** Annual wood growth of *Fraxinus excelsior* L. by stock in various types of forests of the Volga Region (on the example of the Penza region).

Forest Types	Oak Forest Aegopodium	Oak Forest Nettle	Oak Forest Hazel–Linden	Oak Forest Aegopodium-Motley Grass	Oak Forest Carex-Motley Grass
Annual increase in timber stock, m^3^	3.8	3.7	3.6	2.9	2.3

**Table 5:** Parameters of *Fraxinus excelsior* L. stands in various types of forests of the Volga Region (on the example of the Penza region).

Forest Types	Types of Habitat Conditions	Average Height, m	Average Diameter, cm	Completeness	Reserve, m^3^
Oak forest aegopodium	D2 (fresh oak grove)	14.7	17.5	0.7	144.6
Oak forest nettle	D3 (wet oak forest)	16.3	18.2	0.7	161.0
Oak forest hazel–linden	C2 (fresh sudubrava)	12.3	13.7	0.7	114.4
Oak forest aegopodium-motley grass	D2 (fresh oak grove)	15.5	16.5	0.7	148.0
Oak forest carex- motley grass	D1 (dry oak forest)	14.0	14.9	0.8	148.5
Mean		14.6	16.2	0.7	143.3

## Data Availability

The datasets generated and analyzed during the current study are available from the corresponding author on reasonable request.
